# Molecular Mechanisms Underlying α-Linolenic Acid-Enhanced Reproductive Performance in Male Blue Foxes

**DOI:** 10.3390/ani16192984

**Published:** 2026-09-22

**Authors:** Chongshan Yuan, Shuai Qi, Yu Tian, Wenhui Cao, Jia Wang, Min Wu, Gongqing Wei, Xingyuan Zhu, Xinyan Cao, Aiwu Zhang

**Affiliations:** Key Laboratory of Animal Production, Product Quality and Security, Ministry of Education, Jilin Agricultural University, Changchun 130118, China; yuanchongshan@jlau.edu.cn (C.Y.); 18642137669@163.com (S.Q.); tianyu221229@163.com (Y.T.); 18634611920@163.com (W.C.); wangjia032900@outlook.com (J.W.); koo331500@163.com (M.W.); wgq6611@126.com (G.W.); 17612431507@163.com (X.Z.)

**Keywords:** reproduction, testis, seminal plasma, transcriptomics, metabolomics

## Abstract

α-Linolenic acid (ALA), an essential n-3 polyunsaturated fatty acid, exerts critical effects on animal reproductive function. However, the molecular mechanisms by which dietary ALA regulates reproductive performance in male blue foxes remain largely unclear. In the present study, 16 male blue foxes of similar age and condition were assigned to four dietary groups (n = 4 per group) receiving a basal diet supplemented with 0, 0.5, 1.25, or 2 g/kg of ALA. q-PCR validation further confirmed that medium-dose ALA supplementation significantly upregulated mRNA levels of steroid synthesis-related genes (*StAR*, *LHCGR*, *INSL3*) and antioxidant gene *GPX3*, while inhibiting the calcium-signaling gene *GRIN1*. Untargeted liquid chromatography–mass spectrometry (LC–MS) metabolomics of seminal plasma and RNA-seq transcriptomics of testicular tissue were combined to explore the underlying molecular pathways of ALA in terms of its modulating male blue fox reproduction. A total of eight significantly altered seminal plasma metabolites were screened out through metabolomics: mainly arachidonic acid, anandamide, oxaloacetic acid, guanine, lactic acid, dihydrodaidzein, stearic acid, and valylisoleucine. Transcriptomic analysis identified seven differentially expressed genes (DEGs), including *FRAS1*, *COL1A1*, *CDH17*, *CLDN4*, *CTH*, *KYAT1*, and *GRIN1*, enriched in three core KEGG pathways: ECM-receptor interaction, cell-adhesion molecules and selenocompound metabolism. Spearman correlation analysis revealed extensive significant correlations between seminal-plasma differential metabolites and testicular genes, indicating complicated metabolite-gene crosstalk. Collectively, appropriate dietary ALA remodels the seminal-plasma metabolic microenvironment, stabilizes testicular tissue structure, enhances gonadal antioxidant capacity and facilitates testicular steroidogenesis, thereby improving the reproductive potential of male blue foxes. This study elucidates the nutritional regulatory mechanism of ALA in breeding male blue foxes and provides theoretical references for the application of ALA as a functional feed additive in the production of canines.

## Simple Summary

Alpha-linolenic acid (ALA) is an indispensable nutrient for animals, but how it affects the reproduction of male blue foxes remains poorly understood. This study investigated the effects of dietary ALA supplementation on male blue fox reproduction with four ALA dosage treatments. The results showed that moderate ALA supplementation effectively boosted the expression of genes related to testicular hormone synthesis and antioxidant capacity, while regulating key calcium-signaling genes. Combined metabolomic and transcriptomic analysis identified key differential metabolites in seminal plasma and functional genes in testes. Our results explain why ALA improves semen quality in male blue foxes and support its use as a feed additive. Since blue foxes belong to the same animal family as dogs, these findings may also help improve reproductive health in canines.

## 1. Introduction

Blue foxes (*Vulpes lagopus*) are among the most economically significant fur-bearing animals worldwide, with commercial farming operations established across Northern Europe, Asia, and North America [1]. However, the litter size, survival, and offspring quality of fox-production systems are heavily contingent upon reproductive efficiency. In the wild, changes in the physiological state of canines can influence population communication, and it is easy to affect the reproductive performance of foxes during artificial breeding [2,3]. In commercial fox-breeding operations, artificial insemination is widely employed, and a single male may be responsible for inseminating numerous females within a breeding season [4]. Consequently, suboptimal semen quality or diminished reproductive capacity in male foxes can substantially impair conception rates, litter sizes, and overall reproductive outcomes [5]. Nutritional imbalance, oxidative stress and insufficient gonadal hormone secretion are the primary contributors to deteriorating male fertility [6]. Polyunsaturated fatty acids (PUFAs) are essential dietary components that play a critical role in maintaining male reproductive function, largely through their influence on sperm-membrane integrity, antioxidant defense systems, and endocrine regulation of the hypothalamic-pituitary-gonadal axis [7]. Our previous study demonstrated that dietary supplementation with α-linolenic acid (ALA) significantly improved semen quality, antioxidant capacity, and testicular histomorphology in blue foxes [8]. However, further exploration of the potential molecular mechanisms by which ALA regulates reproductive performance through multi-omics approaches, such as transcriptomics and metabolomics, is needed.

As the medium for spermatozoa, seminal plasma provides essential nutrients and signaling molecules that modulate sperm motility, viability, and fertilization potential [9]. Given that alterations in seminal-plasma metabolite profiles are closely associated with compromised sperm quality and subfertility in numerous mammalian species, the identification of reliable metabolic biomarkers has emerged as a promising approach for the prediction of male fertility potential [10]. Metabolomics technology provides the possibility of analyzing biomarkers related to semen quality [11]. Studies in various species, including cattle [12], pigs [13], and poultry [14], have demonstrated that specific metabolites, such as carnitines, amino acids, and lipids, are differentially abundant in the seminal plasma of individuals with contrasting fertility phenotypes. However, despite the growing body of evidence in livestock species, comprehensive metabolomic characterization of seminal plasma in foxes remains limited. Testis serves as the core organ governing spermatogenesis and androgen synthesis, where coordinated transcriptional regulation orchestrates germ-cell proliferation, meiosis and sperm maturation [15]. Spermatogenesis is a highly coordinated and complex biological process that is precisely regulated by the coordinated expression of thousands of genes [16]. Identifying key genes and understanding their regulatory mechanisms during testicular development and spermatogenesis is, therefore, essential for improving reproductive efficiency in animal-production systems [17]. In livestock species, transcriptomic analyses have been successfully applied to investigate testicular development under various nutritional [18], environmental [19], and pathological conditions [20], revealing critical genes and signaling pathways that regulate spermatogenesis and steroidogenesis. However, the molecular mechanisms governing testicular development and male fertility in foxes remain poorly understood.

Based on our previous finding that dietary ALA improves reproductive performance in blue foxes, we hypothesize that this enhancement is driven by coordinated changes in testicular gene expression and seminal-plasma metabolite profiles. We collected samples from previous animal studies and used an integrated transcriptomic and metabolomic approach to identify the specific molecular pathways and key biomarkers that mediate this effect. We further hypothesize that this multi-omics analysis will yield a set of candidate genes and metabolites that can serve as reliable predictive markers for the fertility of canines.

## 2. Materials and Methods

### 2.1. Animal Management and Experimental Design

In our previous study, a total of 40 healthy, 7-month-old male blue foxes of similar age and condition were randomly assigned to four dietary groups (n = 10 per group) receiving a basal diet (Table 1) supplemented with 0, 0.5, 1.25, or 2 g/kg of ALA. The ALA product (purity > 95%) was provided by Jiangsu Baoshijia Biotechnology Co., Ltd. (Nanjing, China). ALA was mixed into the basal diet, and stored at 4 °C prior to use. Diets were prepared fresh weekly and kept at 4 °C in sealed, opaque containers to minimize oxidation. The results showed that dietary supplementation with ALA in male blue foxes enhances semen-quality parameters including motility, viability, and concentration, while reducing the sperm abnormality rate [8]. Therefore, based on the preliminary experimental animals, this study randomly selected 4 blue foxes from each group for transcriptome and metabolome analysis. The study was conducted at Institute of Special Animal and Plant Sciences of Chinese Academy of Agricultural Sciences, Zuojia, Jilin Province, China (44°01′ N, 126°54′ E) from December 2024 to March 2025. During the experimental period, the natural photoperiod was approximately 12 h light:12 h dark (sunrise 05:30–06:00 and sunset 17:30–18:00), with ambient temperatures ranging from 8 °C to 18 °C, which corresponds to the late breeding-preparation phase and early mating season for blue foxes in this region. The experiment consisted of a 1-week adaptation period followed by a 10-week formal feeding period. All foxes were individually housed in stainless-steel cages (1.0 m × 1.0 m) under identical management conditions, with ad libitum access to water, and fed twice daily (06:30 and 16:00). After the 10-week feeding period, semen was collected from each male fox twice using a sterile 50 mL semen-collection cup. The collected semen samples were immediately centrifuged, and the seminal plasma was isolated and stored at −80 °C for subsequent metabolomic analysis. Following semen collection, the foxes were slaughtered, and the testes were rapidly excised. The testicular tissue was immediately snap-frozen in liquid nitrogen and stored at −80 °C for subsequent transcriptomic sequencing analysis [18]. All animal procedures were performed in accordance with the Guidelines for the Care and Use of Laboratory Animals. The study protocol was approved by the Institutional Animal Care and Use Committee of Jilin Agricultural University (20240816004). The detailed experimental procedures and sample processing were performed exactly as described in our previous study [8].

### 2.2. q-PCR Validation

Total RNA was extracted from testicular tissue samples using TRIzol reagent (Invitrogen, Carlsbad, CA, USA), according to the manufacturer’s instructions. RNA purity and concentration were assessed using a NanoDrop 2000 spectrophotometer (Thermo Fisher Scientific, Waltham, MA, USA). The reaction system (20 μL) contained 10 μL SYBR Green Master Mix, 0.4 μL forward primer, 0.4 μL reverse primer, 2 μL diluted cDNA template, and 7.2 μL nuclease-free water (Table 2). q-PCR was performed using TB Green^®^ Premix Ex Taq™ II (Takara Bio, Shiga, Japan) on a LightCycler^®^ 480 q-PCR System (Roche Diagnostics, Basel, Switzerland). The cycling conditions were as follows: initial denaturation at 95 °C for 30 s, followed by 40 cycles of 95 °C for 5 s and 60 °C for 30 s. Each reaction was performed in triplicate for each biological replicate. The relative expression levels of target genes were calculated using the 2^−ΔΔCt^ method, with β-actin serving as the internal reference gene.

### 2.3. cDNA Library Construction and Illumina Sequencing

Total RNA was isolated from frozen tissue using TRIzol (Invitrogen, Carlsbad, CA, USA) reagent, following the manufacturer’s standard protocol. Sequencing libraries were constructed using the Illumina TruSeq Stranded mRNA Library Prep Kit (Illumina, San Diego, CA, USA). Briefly, mRNA was enriched from total RNA by oligo(dT) magnetic beads, followed by fragmentation and first-strand and second-strand cDNA synthesis. End repair, A-tailing, and sequencing adapter ligation were performed sequentially. Fragments of 200–300 bp were purified via agarose gel electrophoresis, enriched by PCR amplification, and quantified for library quality control. The libraries were then sequenced on an Illumina NovaSeq 6000 platform. Raw FASTQ sequencing data were processed with Fastp software (version 1.2.0) to remove low-quality reads.

### 2.4. DEG Screening and Functional-Enrichment Analysis

High-quality clean reads were aligned to the fox reference genome using HISAT2 software (version 2.2.3). Differential expression analysis between groups was performed using the DESeq2 package (version 1.48.1) in R. Genes with |log2FC| > 1 and *p* < 0.05 were identified as differentially expressed genes (DEGs). Principal component analysis (PCA) was performed on normalized FPKM data. Volcano plots were generated to exhibit the distribution of DEGs based on fold change (FC) and statistical significance. All DEGs were annotated against Gene Ontology (GO) and Kyoto Encyclopedia of Genes and Genomes (KEGG) databases using the cluster Profiler R package.

### 2.5. Liquid Chromatography–Tandem Mass Spectrometry (LC–MS/MS) Analysis

For metabolite extraction, 100 μL semen sample was centrifuged at 12,000 rpm for 20 min at 4 °C. The resulting supernatant was transferred to a new tube and dried under nitrogen flow. Dried residues were reconstituted with 100 μL of 80% methanol solution, and the re-dissolved extracts were stored at −80 °C for subsequent LC–MS/MS analysis. An aliquot (10 μL) from each sample extract was combined to generate pooled quality-control (QC) samples. Untargeted metabolomics analysis was performed using ultra-high-performance liquid chromatography (UHPLC) systems (Thermo Fisher Scientific, USA) coupled to high-resolution mass spectrometers (Thermo Q Exactive HF-X, Bremen, Germany). Chromatographic separation was achieved on a reversed-phase C18 column (ACQUITY UPLC HSS T3, 150 × 2.1 mm, 1.8 μm, Waters Corporation, Milford, MA, USA). Mobile phase A consisted of 5 mM ammonium acetate mixed with 5 mM acetic acid in water, and mobile phase B was pure acetonitrile. The column oven temperature was maintained at 40 °C with a constant flow rate of 0.3 mL/min, and the injection volume was set to 4 μL. A standard-gradient elution program was adopted for metabolite separation. QC samples were inserted every 10 test samples during the whole detection sequence to monitor system stability and data repeatability.

### 2.6. Raw-Data Preprocessing and Metabolite Annotation

Raw LC–MS data were processed using XCMS software (version 4.6.0) for peak detection, alignment, and normalization. Metabolite identification was performed by matching experimental spectra against reference databases. PCA was applied to visualize global metabolic separation between groups and assess clustering of QC samples to verify analytical reliability. PLS-DA was utilized to screen differential metabolites by variable importance in terms of projection (VIP) values, with Q^2^ > 0.5 considered acceptable. Univariate statistical analyses were performed using Wilcoxon rank-sum tests to compare metabolite levels between groups. Metabolites with |log_2_FC| ≥ 1, *p* ≤ 0.05 and VIP ≥ 1 were considered significantly different. Hierarchical clustering heatmaps, volcano plots and Venn diagrams were generated to visualize the abundance patterns and shared/specific differential metabolites across comparison groups. Pearson correlation analysis was conducted to construct metabolite correlation networks, with correlation coefficients |r| > 0.8 and *p* < 0.05 retained for network visualization. Functional enrichment of screened differential metabolites was implemented based on the KEGG metabolic-pathway database.

### 2.7. Spearman Correlation Analysis

To investigate the potential regulatory relationships between seminal-plasma metabolites and testicular gene expression, Spearman’s rank correlation analysis was performed. Correlation coefficients were calculated using R software (version 4.2.1), and statistical significance was determined at *p* < 0.05. The resulting correlation matrix was visualized as a heatmap to display the strength and direction of associations between metabolites and genes.

## 3. Results

### 3.1. Validation of DEGs by q-PCR

To investigate the effect on reproductive performance of adding ALA to the diet, the expression levels of genes were examined by referring to q-PCR in testicular tissues from the control and ALA-supplemented groups (Figure 1). The relative expression levels of *StAR*, *GPX3*, *INSL3*, and *LHCGR* were significantly upregulated and *GRIN1* was significantly down regulated in the Z2 group compared with the control group (*p* < 0.05).

### 3.2. Transcriptomic Profiling and Identification of DEGs

Clear segregation was observed between the control and each ALA addition group in the PCA score plots (Figure 2A,D,F,H). The Venn diagram illustrates the overlap of DEGs among the pairwise comparisons (Figure 2B). A total of 32 common DEGs were shared across all four comparison sets. Quantitative statistics of DEGs demonstrated a clear dose-dependent trend (Figure 2C). Volcano plots were further applied to visualize the overall distribution of DEGs for each pairwise contrast (Figure 2E,G,I).

### 3.3. Functional-Enrichment Analysis of DEGs in Testicular Tissue

In the D vs. Z1 comparison, DEGs were significantly enriched in terms of cellular components such as extracellular region and cell periphery, while molecular functions were dominated by tissue development. Key biological processes included peptidase inhibitor activity (Figure 3A). For the D vs. Z2 comparison, significant enrichments were observed in terms of plasma membrane and cell periphery, with biological processes highlighting transmembrane transport and tissue development (Figure 3C). In the D vs. Z3 comparison, cell periphery, extracellular region and plasma membrane remained prominent, alongside specific biological processes such as response to external stimulus and defense response (Figure 3E). KEGG analysis revealed distinct pathway alterations across dosage groups. In the D vs. Z1 comparison, pathways related to ECM-receptor interaction, neuroactive ligand-receptor interaction, and thyroid hormone synthesis were significantly enriched (Figure 3B). The D vs. Z2 comparison highlighted cell-adhesion molecules, aldosterone synthesis and secretion, and drug metabolism-cytochrome P450 (Figure 3D). Notably, the D vs. Z3 comparison showed strong enrichment in terms of cell-adhesion molecules, calcium-signaling pathway, and PPAR-signaling pathway (Figure 3F).

### 3.4. Screening of Key DEGs

Functional enrichment of testicular DEGs highlighted three predominant KEGG pathways, namely ECM-receptor interaction, cell-adhesion molecules, and selenocompound metabolism. Seven representative DEGs of D vs. Z2 with highly significant expression changes (*p* < 0.05) were extracted corresponding to these three core pathways (Table 3). Specifically, the core DEGs were *FRAS1*, *COL1A1*, *CDH17*, *CLDN4*, *CTH*, *KYAT1*, and *GRIN1*.

### 3.5. Quality Control and Global Metabolomic Profiling of Seminal Plasma

As shown in Figure 4A,B, the overlaid total ion chromatograms (TIC) of QC samples exhibited high consistency in terms of both peak retention times and intensities under the positive and negative ion modes, respectively. Hierarchical clustering heatmaps based on all detected metabolites revealed distinct separation trends among the four experimental groups (Figure 4C,D). In the positive ion mode (Figure 4C) and the negative ion mode (Figure 4D), metabolites with similar abundance patterns were clustered together according to the left-side dendrogram. Distinct abundance signatures were observed between the control group and ALA supplementation groups, and obvious separation existed among the Z1, Z2 and Z3 treatments, indicating that dietary ALA intervention reshaped the metabolic profiles of blue fox seminal plasma. Pearson correlation-coefficient-matrix heatmaps were further generated to assess sample repeatability and intergroup differentiation (Figure 4E,F). High intra-group correlation coefficients were observed within each treatment group, demonstrating biological repeatability among the replicates per group.

### 3.6. Multivariate Statistical Analysis and Identification of Differential Metabolites

PCA and partial least-squares discriminant analysis (PLS-DA) were performed to visualize the global metabolic separation of seminal-plasma samples between the groups under both the positive and negative ion modes (Figure 5A–P). The PCA score plots demonstrated a clear separation trend between the groups under both the positive and negative ion modes, indicating the distinct metabolic profiles among the treatments. Consistent with the PCA outcomes, clear segregation was observed between the control and each ALA addition group in the PLS-DA score plots across both the ion detection modes. Univariate statistical analysis was conducted using volcano plots (Figure 5Q–V). In the comparison between the control and ALA-supplemented groups, a substantial number of metabolites were significantly upregulated (red dots) or downregulated (blue dots).

### 3.7. KEGG Pathway-Enrichment Analysis of Differential Metabolites

In the multi-group comparison (D vs. Z1 vs. Z2 vs. Z3), protein digestion and absorption and ABC transporters emerged as the most significantly enriched pathways in both the positive and negative ion modes, exhibiting the highest rich factors and lowest *p*-values (Figure 6A,B). In the comparison between the control group (D) and the low-dose ALA group (Z1), protein digestion and absorption and ABC transporters remained the dominant pathway (Figure 6C,D). As the ALA dosage increased to the medium level (Z2), D-amino acid metabolism and the citrate cycle (TCA cycle) became notably enriched, alongside the central carbon metabolism pathways (Figure 6E,F). In the high-dose group (Z3), compared to the control, ABC transporters and purine metabolism were prominently enriched (Figure 6G,H).

### 3.8. Identification of Core Differential Metabolites

Differential metabolites of D vs. Z2 were screened following unified statistical thresholds: VIP > 1, false discovery rate (FDR) < 0.05, |FC| > 1.2 and *p* < 0.05. A total of eight significantly upregulated metabolites satisfying all criteria were identified from the seminal plasma of male blue foxes receiving dietary ALA supplementation (Table 4). Specifically, the eight core differential metabolites were arachidonic acid, anandamide, oxaloacetic acid, guanine, lactic acid, dihydrodaidzein, stearic acid and valylisoleucine.

### 3.9. Correlation Analysis Between Differential Metabolites and DEGs

As shown in Figure 7, guanine displayed the strongest positive correlation with *FRAS1*. Dihydrodaidzein and lactic acid were significantly positively correlated with most detected genes. Valylisoleucine showed a significant positive correlation with *CLDN4*, and arachidonic acid maintained weak positive correlations with all tested genes without statistical significance. Stearic acid presented widespread significant negative correlations with the six DEGs. Anandamide showed significant negative correlations with *FRAS1*, *COL1A1*, *CDH17* and *CTH*. Oxaloacetic acid was negatively correlated with most genes, and reached significance for *COL1A1* and *CLDN4*.

## 4. Discussion

Our previous study found that dietary ALA supplementation improves reproductive performance in male blue foxes [8]. However, the molecular drivers of these improvements remained unclear. To bridge this gap, we performed an in-depth analysis using biological samples collected from that same experiment, specifically combining seminal-plasma metabolomics with testicular transcriptomics. The results confirmed that appropriate ALA supplementation remodels the seminal-plasma metabolic microenvironment and regulates testicular gene expression related to tissue structure, antioxidant capacity and steroid synthesis. The present findings advance our understanding of ALA-mediated reproductive regulation in male blue foxes and provide theoretical guidance for optimizing nutritional strategies for breeding canines.

Based on our previous feeding trial identifying 1.25 g/kg as the optimal dietary ALA supplementation level for improving reproductive traits in male blue foxes, the present q-PCR assay was performed to dissect the molecular basis through which ALA regulates testicular function and reproductive performance. The coordinated elevation of *LHCGR* and *StAR* enhanced luteinizing hormone signal transduction and cholesterol transport in Leydig cells, accelerating testosterone synthesis [21,22]. Elevated *INSL3* further confirmed well-maintained leydig cell activity, which inhibits germ-cell apoptosis and stabilizes meiotic progression, ultimately reducing sperm-malformation rate [23]. Upregulated *GPX3* reinforced testicular antioxidant defense to eliminate excess reactive oxygen species and relieve oxidative injury to spermatogenic cells [24]. As a key mediator of calcium signaling, the reduction in *GRIN1* implied that optimal ALA supply may restrict calcium-triggered germ-cell apoptosis [25]. In summary, optimal 1.25 g/kg dietary ALA remodels the expression of steroidogenic, anti-oxidative and spermatogenesis-related genes in male fox testes.

We further analyzed the mechanism of ALA in regulating the reproductive performance of blue foxes using transcriptome analysis. Previous studies have demonstrated that dietary fatty-acid supplementation can alter the lipid composition of testicular cell membranes [26]. In this study, the enrichment of transmembrane transport processes suggests that ALA may modulate the transport of nutrients, ions, and metabolites across cellular membranes in testicular cells, potentially affecting the biochemical microenvironment essential for germ-cell development [27]. In this study, the enrichment of calcium-signaling and steroid-hormone-biosynthesis pathways underscores the endocrine-modulatory effects of ALA. Calcium signaling is indispensable for sperm motility, capacitation, and the acrosome reaction, while local steroidogenesis is a prerequisite for maintaining spermatogenesis [28]. Previous metabolomic studies in bulls have confirmed that seminal-plasma metabolites and testicular gene networks are tightly correlated with sperm motility and oxidative phosphorylation [29]. ECM-receptor interaction and cell-adhesion-molecule pathways constitute the structural foundation of an intact spermatogenic microenvironment [30,31]. *FRAS1* and *COL1A1* encode key extracellular matrix components that maintain the integrity of seminiferous tubule basement membrane [32,33]. Meanwhile, *CDH17* and *CLDN4* are pivotal tight and adherens junction proteins governing cell–cell contact between somatic and germ cells [34,35]. The selenocompound metabolism pathway, represented by upregulated *CTH* and *KYAT1*, undertakes critical antioxidant functions in testicular tissue [36,37]. Taken together, dietary ALA simultaneously optimizes testicular tissue’s structural stability, intercellular material exchange and gonadal antioxidant-defense capacity.

Afterwards, we analyzed the overall changes in the metabolites of seminal plasma. Both PCA and PLS-DA score plots revealed clear separation between the D group and other groups. These findings align with previous reports demonstrating that dietary fatty-acid composition can modulate the metabolic profiles of seminal plasma in bulls [38]. In the present study, protein digestion and absorption and ABC transporters emerged as the most significantly enriched pathways across multiple comparison groups. The prominent enrichment of these two core pathways indicated that dietary ALA fundamentally modulates the substance transport and protein metabolic homeostasis of seminal plasma, which is essential for maintaining the sperm’s normal nutritional supply and structural integrity [39]. With the increase in ALA dosage to the medium level, key energy metabolic pathways including D-amino acid metabolism and the citrate cycle (TCA cycle) were significantly enriched, suggesting that moderate ALA supplementation further optimizes the central carbon metabolism and energy-supply efficiency of seminal plasma, thereby providing sufficient energy support for the sperm’s motility maintenance and in vitro survival [40]. As a precursor of arachidonic acid, dietary ALA can be elongated and desaturated in the male reproductive tract to synthesize PUFAs that constitute the lipid bilayer of sperm plasma membranes [41]. The increased stearic acid content balances PUFA proportions in seminal plasma and alleviates excessive lipid-peroxidation risk induced by high unsaturated fatty-acid concentrations [42]. Elevated lactic acid acts as a rapid energy donor for short-term sperm movement, whereas oxaloacetic acid replenishes the TCA cycle pool to maintain sustained mitochondrial energy production [43,44]. The sharp rise of guanine in ALA-treated seminal plasma indicates that ALA nutrition reduces reactive oxygen species-induced DNA fragmentation, alleviating asthenospermia and teratozoospermia caused by genetic damage [45]. Metabolites showing strong positive correlations with ECM and cell-adhesion-related genes (*FRAS1*, *COL1A1*, *CDH17*, and *CLDN4*) such as guanine, lactic acid and dihydrodaidzein may act as signaling molecules supporting the integrity of seminiferous tubule structures and the blood–testis barrier function [46]. Lactic acid, the primary glycolytic energy substrate, was positively correlated with selenocompound metabolic genes *CTH* and *KYAT1*. Sufficient energy supply in seminal plasma likely supports the antioxidant system, which is mediated by selenium metabolism, jointly maintaining spermatogenic homeostasis [47]. However, the sample size of the group for analysis may reduce the statistical power and limit generalizability in this study such that future studies with larger cohorts and multi-batch validation are warranted.

## 5. Conclusions

In conclusion, these results demonstrate that appropriate ALA supply improves the reproductive capacity of male blue foxes by optimizing the seminal-plasma microenvironment and testicular physiological function. These identified metabolites and genes hold promise as potential biomarkers for canines, enabling breeders to monitor reproductive status and optimize breeding decisions in real-world production settings.

## Figures and Tables

**Figure 1:**
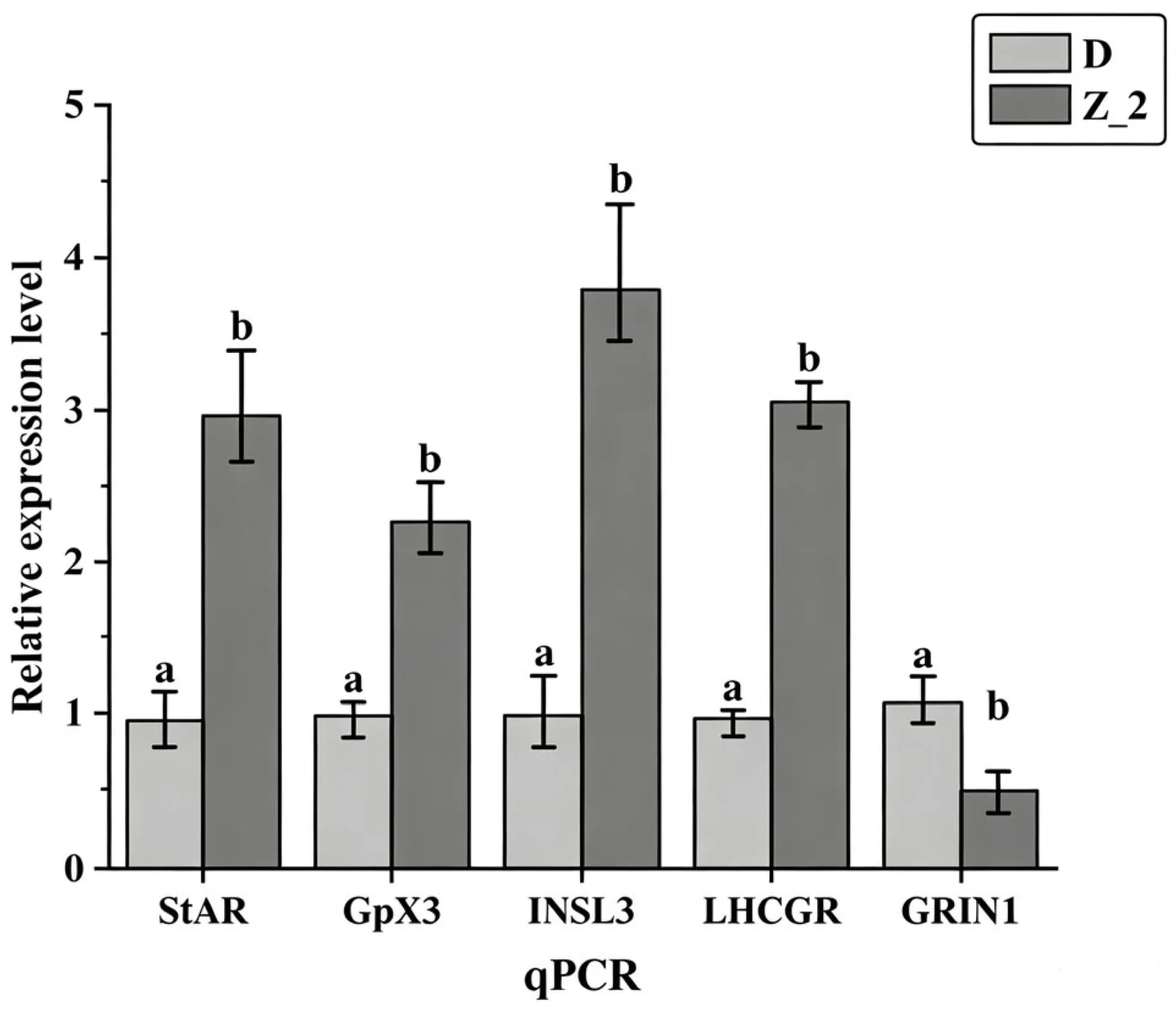
Relative mRNA expression of genes in testicular tissue (n = 4). D: control group and Z2: 1.25 g/kg ALA. Values are means ± SD. Different lowercase letters denote significant differences (*p* < 0.05).

**Figure 2:**
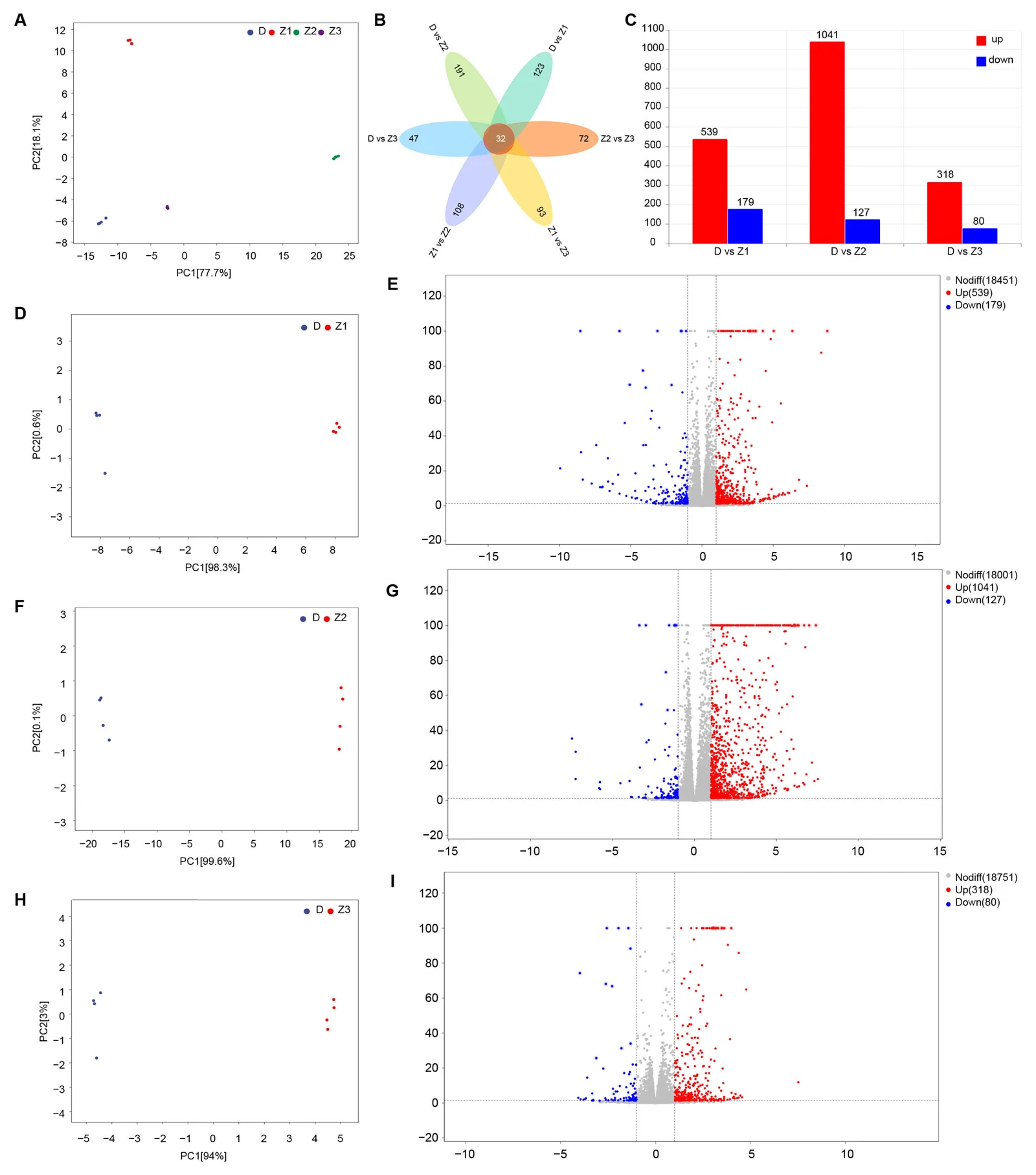
Transcriptome profiling and differential-gene-expression analysis of testicular tissue (n = 4). (**A**) PCA of testicular transcriptome samples across all four experimental groups. (**B**) Venn diagram illustrating the overlap of DEGs identified from pairwise comparisons between the control group and each ALA supplementation group. (**C**) Statistical summary of the total number of significantly upregulated and downregulated DEGs screened from each pairwise comparison. (**D**,**F**,**H**) Pairwise PCA score plots for transcriptomic profiles of D vs. Z1, D vs. Z2, and D vs. Z3 comparisons, respectively. (**E**,**G**,**I**) Volcano plots showing the differential expression patterns of testicular genes for D vs. Z1, D vs. Z2, and D vs. Z3 comparisons, respectively.

**Figure 3:**
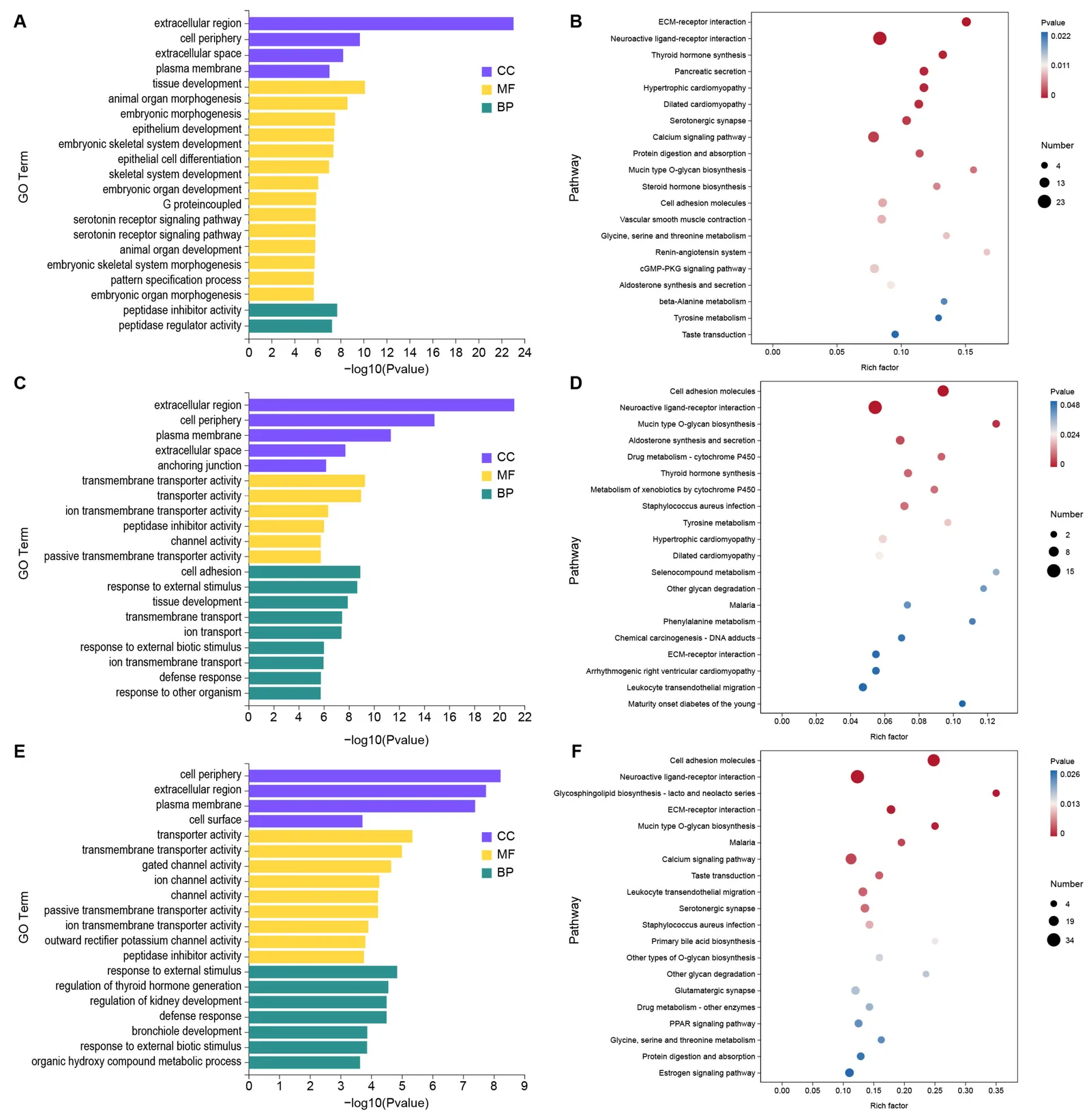
GO and KEGG functional-enrichment analysis of DEGs in testicular tissue (n = 4). (**A**,**C**,**E**) GO enrichment analysis of DEGs for the comparisons of D vs. Z1, D vs. Z2, and D vs. Z3, respectively. GO terms cover three main categories: biological process (BP), cellular component (CC), and molecular function (MF). (**B**,**D**,**F**) KEGG pathway-enrichment analysis of DEGs for the comparisons of D vs. Z1, D vs. Z2, and D vs. Z3, respectively.

**Figure 4:**
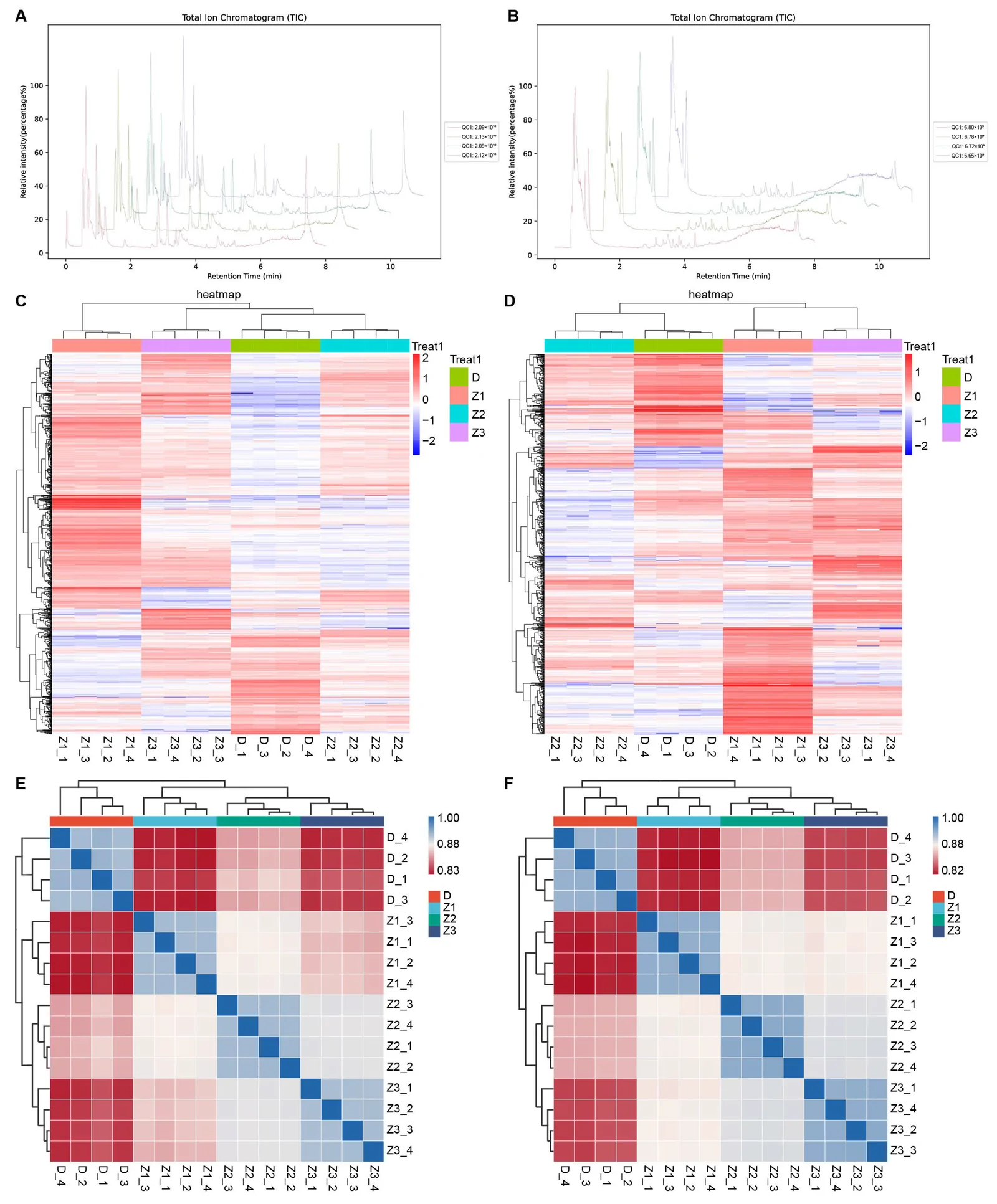
Quality control and overall metabolome profiling (n = 4). (**A**,**B**) Overlay total ion chromatograms (TIC) of quality-control (QC) samples under positive ion mode (**A**) and negative ion mode (**B**). (**C**,**D**) Hierarchical clustering heatmaps of all detected metabolites in positive ion mode (**C**) and negative ion mode (**D**). (**E**,**F**) Pearson correlation-coefficient-matrix heatmaps of all test samples in positive ion mode (**E**) and negative ion mode (**F**).

**Figure 5:**
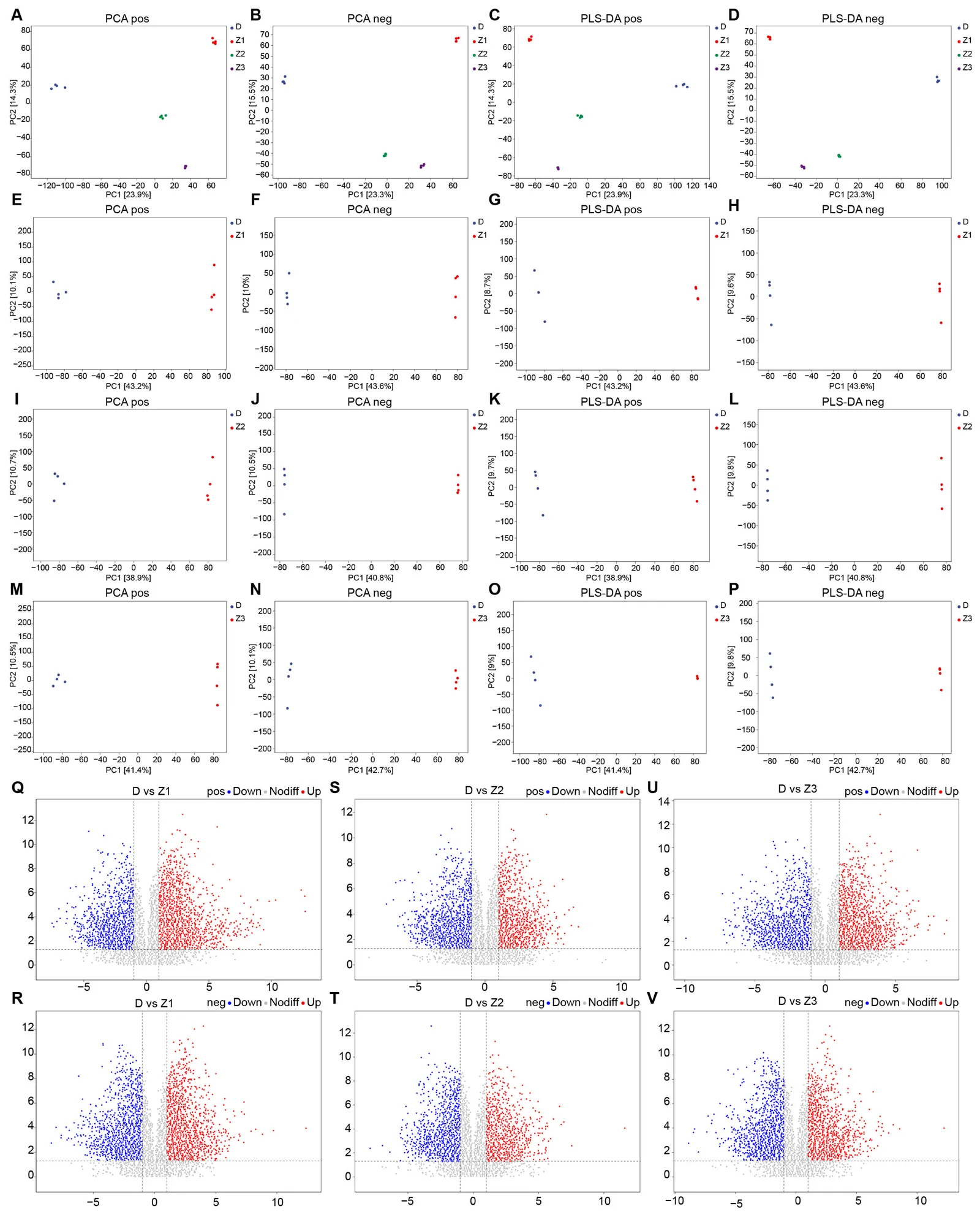
Multivariate statistical analysis and volcano plot (n = 4). (**A**–**P**) PCA and PLS-DA score plots of seminal-plasma metabolites under positive ion mode (pos) and negative ion mode (neg). (**A**,**E**,**I**,**M**) PCA score plots in positive ion mode for comparison between four groups, D vs. Z1, D vs. Z2 and D vs. Z3, respectively. (**B**,**F**,**J**,**N**) PCA score plots in negative ion mode for comparison between four groups, D vs. Z1, D vs. Z2 and D vs. Z3, respectively. (**C**,**G**,**K**,**O**) PLS-DA score plots in positive ion mode for comparison between four groups, D vs. Z1, D vs. Z2 and D vs. Z3, respectively. (**D**,**H**,**L**,**P**) PLS-DA score plots in negative ion mode for comparison between four groups, D vs. Z1, D vs. Z2 and D vs. Z3, respectively. (**Q**–**V**) Volcano plots showing differential metabolites between the control group and each ALA supplementation group. (**Q**,**S**,**U**) Volcano plots under positive ion mode for D vs. Z1, D vs. Z2 and D vs. Z3 comparisons, respectively. (**R**,**T**,**V**) Volcano plots under negative ion mode for D vs. Z1, D vs. Z2 and D vs. Z3 comparisons, respectively.

**Figure 6:**
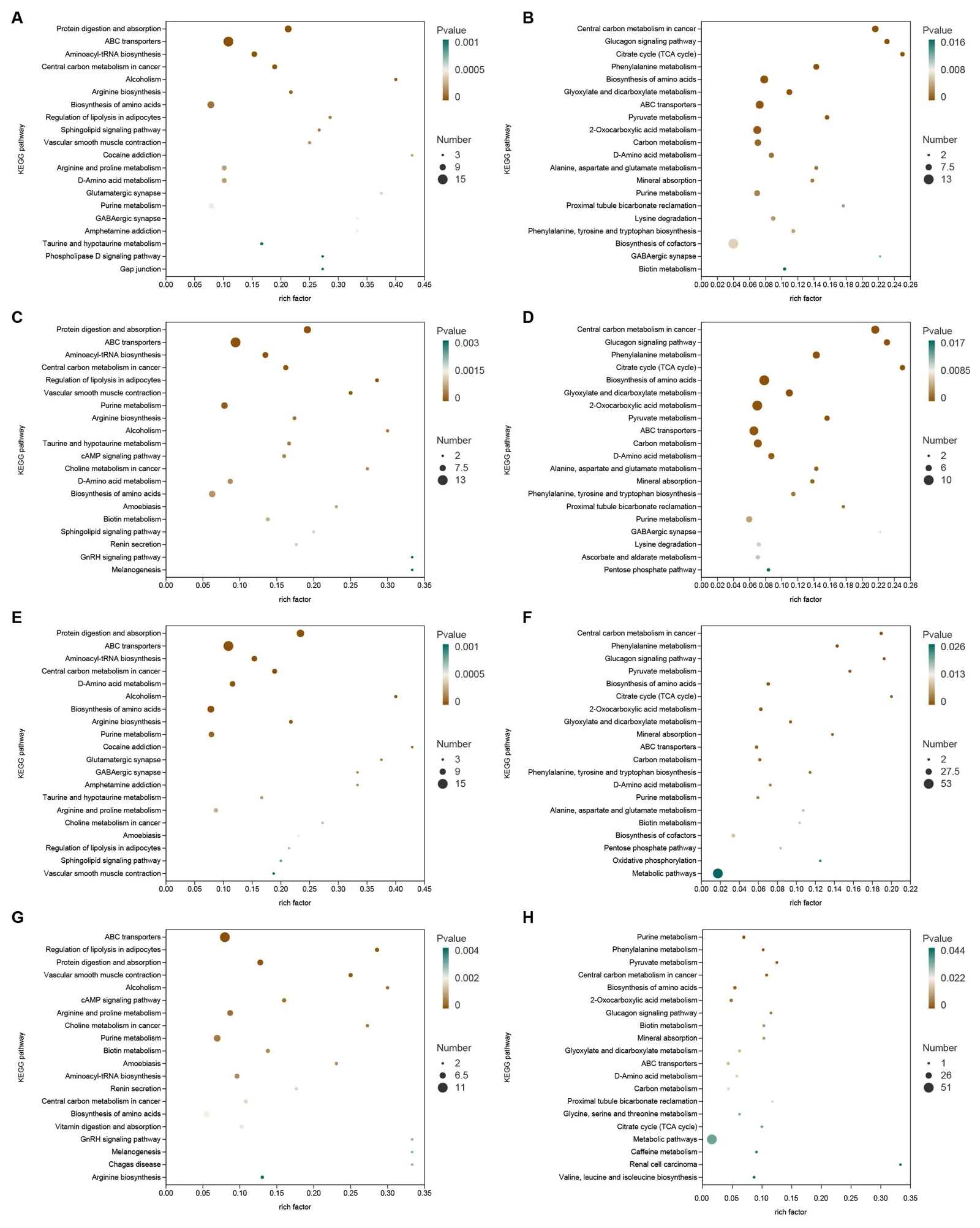
KEGG pathway-enrichment analysis of differential metabolites in seminal plasma (n = 4). (**A**–**H**) Bubble plots illustrating the top 20 significantly enriched KEGG pathways for different comparison groups. (**A**,**C**,**E**,**G**) represent positive-ion-mode analyses for comparison between four groups, D vs. Z1, D vs. Z2, and D vs. Z3, respectively and (**B**,**D**,**F**,**H**) represent negative-ion-mode analyses for comparison between four groups, D vs. Z1, D vs. Z2, and D vs. Z3, respectively.

**Figure 7:**
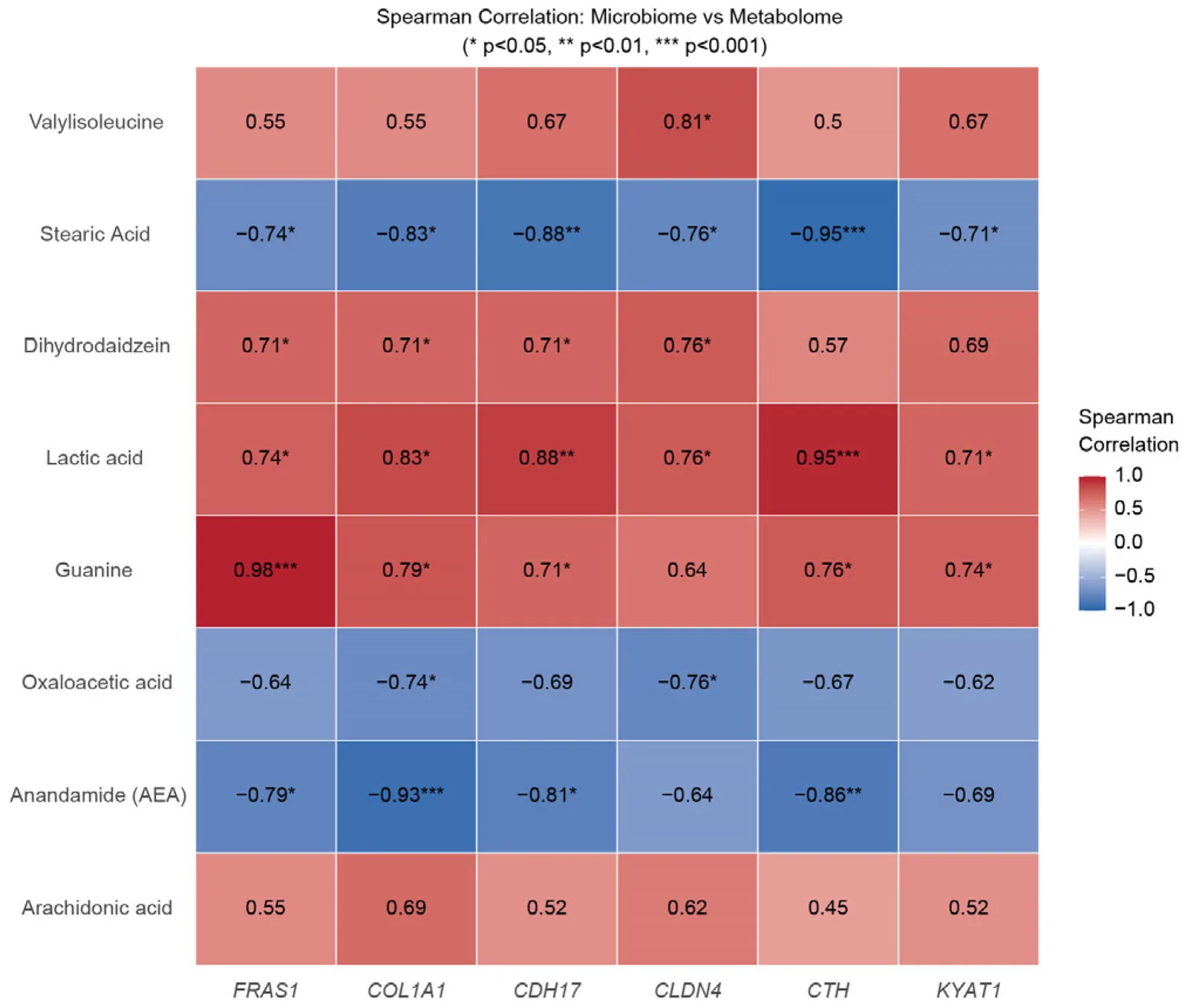
Spearman correlation heatmap between seminal-plasma differential metabolites and testicular DEGs (n = 4). Red indicates positive correlation, and blue indicates negative correlation. * *p* < 0.05, ** *p* < 0.01, and *** *p* < 0.001.

**Table 1:** Composition and nutrient levels of basal diet (dry-matter basis).

Ingredients	Content (%)	Nutrition	Content (%)
Extruded corn	25.05	Crude protein (CP)	28.02
Soybean meal	25.3	Ether extract (EE)	12.54
Bone and meat meal	8.25	Ash	7.29
Corn germ meal	20.75	Ca	1.28
Fish meal	9.2	P	1.02
Soybean oil	10	Methionine	0.49
NaCl	0.3	Lysine	1.66
Premixa	1		
Methionine	0.05		
Lysine	0.1		
Total	100		

**Table 2:** Gene primer sequences.

GeneID	Gene Primer Sequences	Amplicon Length (bp)	Transcriptome ID
StAR	F: GGGAGAGTGGAACCCGAATG R: CCACCAGGTTTCCTGCTGAT	128	ENSVVUG00000020188
GpX3	F: TGAAGGTCCATGACATCCGC R: GTACCAGCGCATGATGGGTA	115	ENSVVUG00000007074
INSL3	F: CCATGCTGGTTCTCGTTCCA R: CAGTAGTGTGCGGGATTGGT	133	ENSVVUG00000010239
LHCGR	F: TTATCGCCATGTTGCCCCTT R: TCTACATCCATGGGGAGGCA	142	ENSVVUG00000005772
GRIN1	F: CCAGTCAAGAAGGTGATCTGCA R: TTCATGGTCCGTGCCAGCTTGA	156	ENSVVUG00000029460
β-Actin	F: GATGAGCTGTCTGCCTTGGT R: GTGGTGCCAGATCCCCTGAT	109	_

**Table 3:** Differential Genes.

Gene ID	Gene Name	log2 FC	*p*-Value	Regulation	KEGG Pathway
ENSVVUG00000024813	*FRAS1*	1.7497	1 × 10^−100^	Up	ECM-receptor interaction
ENSVVUG00000004354	*COL1A1*	1.3607	1 × 10^−100^	Up	ECM-receptor interaction
ENSVVUG00000002787	*CDH17*	5.3991	1 × 10^−100^	Up	Cell-adhesion molecules
ENSVVUG00000024825	*CLDN4*	4.4606	1 × 10^−100^	Up	Cell-adhesion molecules
ENSVVUG00000007214	*CTH*	3.4449	1 × 10^−100^	Up	Selenocompound metabolism
ENSVVUG00000026017	*KYAT1*	2.993	1 × 10^−100^	Up	Selenocompound metabolism
ENSVVUG00000029460	*GRIN1*	−1.051	3.42 × 10^−13^	Down	Calcium-signaling pathway

**Table 4:** Core Differential Metabolites.

ID	Name	FC	*p*-Value	FDR	VIP	Regulation
MP13885.pos	Arachidonic acid	2.56344	3.36 × 10^−8^	6.16 × 10^−6^	1.601248	Up
MP15231.pos	Anandamide	1.853395	1.32 × 10^−7^	1.38 × 10^−5^	1.601092	Up
MN3276.neg	Oxaloacetic acid	1.714464	2.83 × 10^−6^	8.94 × 10^−5^	1.550338	Up
MP4304.pos	Guanine	5.372502	6.29 × 10^−8^	8.69 × 10^−6^	1.602566	Up
MN1058.neg	Lactic acid	2.827241	5.49 × 10^−9^	1.76 × 10^−6^	1.563268	Up
MP2622.pos	Dihydrodaidzein	2.426187	1.83 × 10^−9^	1.55 × 10^−6^	1.603479	Up
MP12868.pos	Stearic Acid	1.220291	6.07 × 10^−7^	3.74 × 10^−5^	1.596234	Up
MN9821.neg	Valylisoleucine	2.46387	1.01 × 10^−10^	2.54 × 10^−7^	1.564725	Up

## Data Availability

The metabolomic data generated in this study have been deposited in the OMIX database (China National Center for Bioinformation) under accession number OMIX020305. The transcriptomic raw sequencing data have been deposited in the NCBI Sequence Read Archive (SRA) under BioProject accession number PRJNA1524480. All other data and materials are available from the corresponding author upon reasonable request.
